# Examination of Brain

**Published:** 1875-06

**Authors:** Jos. G. Richardson

**Affiliations:** Microscopist to the Pennsylvania Hospital, Philadelphia, Pa.


A MONTHLY RECORD OF

*Medicine, Surgery and the Collateral Sciences.*

EDITED BY J. ADAMS ALLEN, M.D., LL.D.: AND WALTER HAY, M.D

VOL. XXXII. — JUNE, 1875. — No. 6.

### Original Communications.

ARTICLE I.

### EXAMINATION OF BRAIN.

BY JOS. G. RICHARDSON, M.D.,

Microscopist to the Pennsylvania Hospital, Philadelphia, Pa.

The specimen from Dr. Walter Hay's case of Traumatic Tetanus, reached me by Express from Chicago, April 23, 1875, and for an alcohol preparation, arrived in admirable condition.

The medulla oblongata had been cut through obliquely (probably in removing the brain at the autopsy), about three-eighths of an inch below the inferior border of the pons varolii, so that, unfortunately, no portion of the spinal cord was preserved.

Thin transverse sections, cut from the stump of the medulla, and therefore on a plane with part of the roots of the pneumogastrics, were some of them prepared by

Lockhart Clarke's method ;* others by that of Gerlach, with double chloride of gold ;† and still others by Gerlach's second method, with carmine and ammonia. When examined microscopically, under various powers, ranging from 50 to 2,000 diameters, these specimens did not furnish any evidence of pathological alterations of structure, and especially none of "granular" or "fluid disintegration," such as is described by Clarke in his original paper.‡

Sections cut from about the middle of the pons, and prepared in like manner by these several different processes, afforded similar negative results, as did likewise vertical slices of tissue from the right lobe of the cerebrum, and of the cerebellum, examined in glycerin, and also in oil of cloves.

In the typical example, detailed at length by Mr. L. Clarke, in the article above referred to, he expressly states (p. 258): "The medulla oblongata and the upper end of the cord, which give origin to the first cervical nerves, were apparently free from disease. At the second cervical nerves, however, streaks and irregular areas of disintegration were observed in different parts of the gray substance, and particularly around the central canal, on the right side of which was a space of considerable size, containing a finely granular fluid, with the debris of blood-vessels and nerves." In Dr. Hay's case, therefore, the results, as far as obtained or obtainable, tend to corroborate Lockhart Clarke's observation, that no structural changes are visible, on microscopical examination, in the brains of patients dying from tetanus.

Neither Dr. W. H. Dickinson, in his excellent report of a case of Tetanus, before the Royal Medical and Chirurgical Society (Med. Times and Gazette, Oct., 1868, p. 460), nor MM. Arloing and Leon Tripier, in their Experimental and

* Frey Microscope and Microscopical Technology, Transl. New York, 1872, p. 354.

† Stricker's Histology, New Syd. Soc. Transl. London, 1872, vol. ii, p. 344.

‡ Medico-Chirurgical Trans. London, 1865, vol. xlviii, p. 255.

Clinical Researches on Tetanus (Archives de Physiologie normale et Pathologique, Tome III, 1870, p. 235), nor Ranke, in his Physiologico-Chemical Study of Tetanus (for an abstract of which see Prager Vierteljahrschrift fur die Praktische Heilkunde, 1866, 89 er Band, Analekten, S. 106), refer to cases in which lesion of any part of the brain was discovered on post mortem examination.

In conclusion, I would express my regret, first, that unavoidable circumstances prevented the gentlemen making this autopsy from securing at least the upper portion of the medulla spinalis; and, second, that subsequent investigations were necessarily performed upon the specimens after they had passed beyond that fresh condition which alone admits of the best preparation and the most satisfactory research.

